# Autologous Platelet Gel (APG): A Preliminary Evaluation of the Mechanical Properties after Activation with Autologous Thrombin and Calcium Chloride

**DOI:** 10.3390/ma14143941

**Published:** 2021-07-14

**Authors:** Antonio Scarano, Calogero Bugea, Lucia Leo, Pablo Santos de Oliveira, Felice Lorusso

**Affiliations:** 1 Department of Innovative Technologies in Medicine & Dentistry, University of Chieti-Pescara, via dei Vestini 31, 66100 Chieti, Italy; calogerobugea@yahoo.it (C.B.); drlucialeo@hotmail.com (L.L.); drlorussofelice@gmail.com (F.L.); 2 Department of Oral Implantology, Dental Research Division, College Ingà, UNINGÁ, Cachoeiro de Itapemirim, Espirito Santo 29312, Brazil; psoliveiraodonto@yahoo.com.br

**Keywords:** platelet concentrates, growth factors, blood derivates, hemocomponents

## Abstract

The tensional and mechanical behavior of regenerative components, grafts, and blood clots represent an essential condition for the success of bone regeneration protocols. Autologous platelet growth factors represent a useful protocol to enhance the soft and hard tissue healing in several fields of medicine and craniofacial surgery. Different protocols for blood concentrates with and without activation have been proposed in literature. The aim of the present study was to investigate in vitro the mechanical properties of autologous platelet gel (APG) with autologous thrombin and calcium chloride. Materials and Methods: A total of 20 APG samples were evaluated; 10 samples were activated by autologous thrombin and calcium chloride (Group I) and 10 samples were non-activated (Group II). The tensile strength and modulus of elasticity were calculated through a static loading test (Lloyd 30 K, Lloyd Instruments Ltd., Segensworth, UK). Results: Group I (activated) reported a tensile strength of 373.5 ± 14.3 MPa, while Group II showed a significantly lower value of 360.5 ± 16.3 MPa (*p* < 0.05). The Young’s modulus was 145.3 ± 10.4 MPa for Group I and 140.3 ± 15.3 MPa for Group II (*p* < 0.05). Conclusions: The effectiveness of the present in vitro simulation showed that the APG activation protocol is able to increase the mechanical characteristics of the blood derivates and could be clinically useful to enhance regenerative procedures.

## 1. Introduction

Dental implants for the rehabilitation of partially or totally edentulous patients are extensively used, with a high-percentage survival rate in regenerated bone [1,2] or in bone with poor quality [3]. In case of insufficient thickness or height of alveolar bone, it is necessary to use bone regeneration procedures. There is a need for the promotion of safer, more efficient, and more successful surgical procedures for bone regeneration.

The graft characteristics, the growth factors and the cellular response are the main factors for bone defect healing [4,5,6]. Clinically, bone substitutes are generally exposed to biomechanical forces such as compression and tensional and torsional stresses, while the physicomechanical characteristics of the substrate can play a key role in the regenerative processes and space maintenance in vivo [7,8,9].

The clinical relevance of the space maintenance concept is determined by the creation of a regenerative space necessary for the stabilization and protection of blood clots, which constitute the trigger component for defect healing and new bone formation [10,11]. In this context, the local cellular response mediated by citokines (such as interleukin-1β, tumor necrosis factor-α, and interferon-γ) and growth factor release in the early stages of bone healing play a fundamental role for the sustaining of the healing processes of the bone defects [12,13].

Different techniques have been proposed by different authors, such as the inlay technique with rigid mesh [14,15], delayed expansion of atrophic mandible (DEAM) [16], sinus lifting, lateralization of inferior alveolar nerve, etc. Today there is great interest in autologous platelet gel, platelet concentrates (PC), and growth factors. They are extensively used in endodontic [17], oral surgery, in implantology [18] and parodontology [19]. Platelet concentrates (PC) have been widely used in bone regenerative procedures for their autologous origin and they offer a great concentration of platelets, when compared to the concentration in natural blood. PC are a growth factor source able to promote angiogenesis and tissue healing in consideration of the fact that a blood supply is a prerequisite for tissue healing [20]. PC were originally introduced by Marx et al. [20], Anitua [21], and Choukroun [22] in the early 1990s and their applications are increasing in many medical fields, such as esthetic medicine [23], orthopedics [24], wound repair, military drill [25], ophthalmology [26] and, of course, in dentistry. PC have been proposed for soft and hard tissue surgery in many different forms as clots reduce in thick membranes to improve the healing of the damaged tissues and the mechanical/ biological behavior of biomaterials and scaffolds [26,27].

There is a lot of interest in the use of PCs for their growth factor content, (TGF-β, PDGF, EGF, VEGF, FGF, IGF-1) present in the platelet α-granules and implicated in the proliferative and inflammatory phase of tissue healing [28].

Moreover, in literature there have been described several activators of platelet derivates and clots, such as bovine thrombin, Russell’s viper venom, calcium gluconate, batroxobin plus calcium, and calcium chloride (CaCl2) [29,30].

They are obtained through centrifugation of whole blood, which separates the plasma components by density gradient; that is, the leucocytes and platelets from red blood cells (RBCs), to form a buffy coat. All single components in PC confer particular biological properties. In research, different machines are used for the preparation and laboratory settings, therefore the quantity of platelet recovery varies between systems and subsequently not all PC are the same; some are unknown, when the name of the machine and other data such as G-force, speed and time of centrifugation, type of vials and method used for platelet activation are not mentioned in the study. The effectiveness of PC used in various medical fields is mainly determined by the chemical and mechanical properties and the concentration of growth factors [31,32]. Autologous platelet gel has been used for soft tissue augmentation [33] and to promote bone healing [27]. 

The aim of this study was to evaluate the mechanical properties of activated-APG protocol and non-activated APG by thrombin and calcium chloride. The null hypothesis was that no differences of tensile strength and elastic modulus were present between activated the APG group and the non-activated APG group.

## 2. Materials and Methods

The present investigation received the ethical approval of the Inter-Institutional Ethics Committee of Faculdade Ingá, UNINGÁ, PR, Brazil, N°89018318.2.0000.5220. The study was conducted according to the ethical guidelines of the Declaration of Helsinki (https://www.wma.net/wp-content/uploads/2018/07/DoH-Oct2008.pdf, accessed on 15 May 2019) and in observation of the current requirements of Italian law. All patients gave written informed consent. A total of 20 APG samples were used, 10 non-activated APG membranes and 10 APG activated membranes with thrombin and calcium chloride (Ubgen Srl Padova, Italy).

### 2.1. Preparation APG

**Figure 1:**
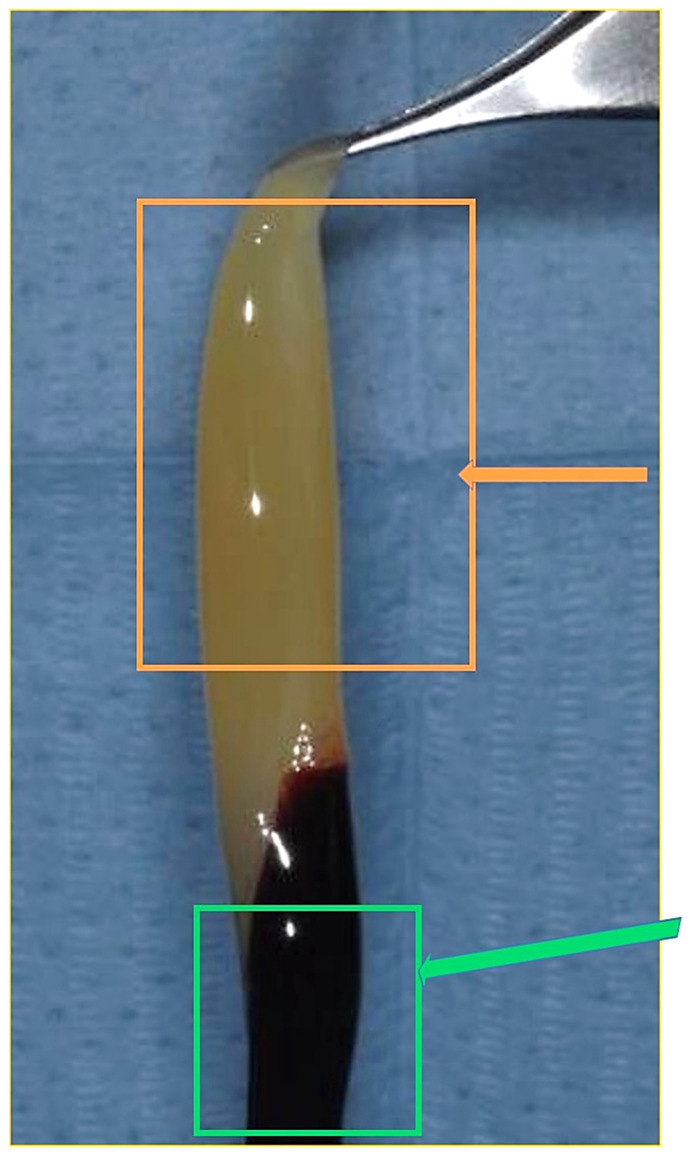
Autologous platelet gel (APG) immediately extracted from the vial appeared composed of two portions, one yellow portion and one red portion. B. The yellow portion (orange arrow) and red portion (green arrow).

**Figure 2:**
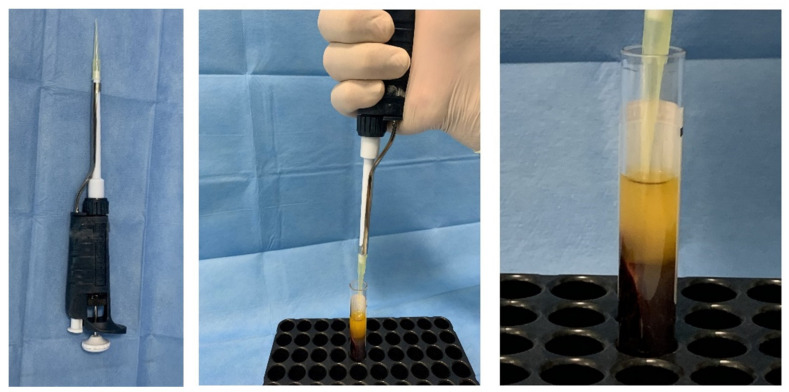
Details of the autologous thrombin separation.

**Figure 3:**
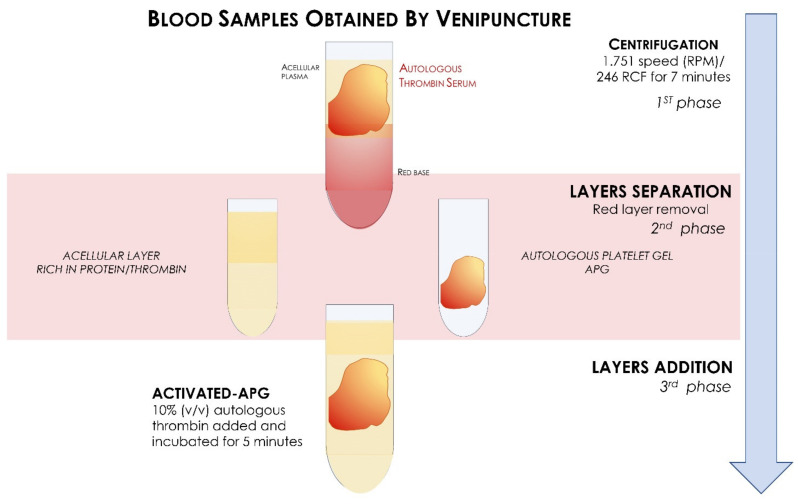
Flow chart of the activated APG formation process.

Blood sampling was carried out on ten adult donors, five men and five women with an average age of 25 ± 7 years, with a negative history as hemorrhagic patients, of alcohol consumption, or anti-coagulant therapy that included a bone regeneration procedure. Also excluded were pregnant and lactating women, patients taking bisphosphonates, and those who had consumed antibiotics within the past 2 months, had diathesis, were smokers, had coagulopathies, and patients under medication, with serious systemic diseases and with non-physiological blood parameters: platelet count out of the range 150,000 to 400,000 per microliter of blood, red cells out of the range 4.7 to 6.1 million cells, or white cell count out of the range 4 × 109/L and 1.1 × 1010/L [34]. An average healthy adult can produce 1011 platelets per day; old platelets are destroyed by phagocytosis in the spleen and liver (Kupffer cells). The number of platelets from donors was mean 274,000 platelets/μL, (range from 140,000 to 351,000) taken from previous blood tests. In oral implantology, blood is usually used for mixed bone grafts to achieve a sticky graft [35], or to produce autologous platelet gel/liquid and then mixed with biomaterials. In the present in vitro investigation, for various cases of oral surgery, the blood was used to obtain autologous platelet liquid. Blood sampling was performed by venipuncture from the median cubital vein, with a 21-G butterfly needle with a preassembled support with Luer Lock attachment, and blood was collected in 9 mL red vacuum vials with a coating of silica activators without anticoagulant or bovine thrombin (Ubgen Srl Padova, Italy). Blood fractionation was performed with a GFONE centrifuge (Ubgen, Vigonza, Padua), with a 1.751 speed (RPM) and relative centrifugal force (RCF) of 246 for 7 min at room temperature. The rpm for centrifugation was achieved with the rotor radius of the centrifuge based on the formula where the rotor radius of the centrifuge used was 5 cm. After centrifugation, each blood sample was divided into three layers: red blood cells on the bottom, plasma in the upper part of the tube, and the autologous platelet gel interposed (Figure 1). The fibrin clot (APG) containing platelets in the middle of the tube, between the red blood cell layer at the bottom and the acellular plasma at the top, was removed from the red tube and the attached red blood cells were discarded and scraped off. A syringe was used to aspirate the acellular layer rich in proteins such as thrombin. For activation, the APG was transferred into a glass and 500 μL of 10% (*v*/*v*) autologous thrombin aspirated from the top layer of the red vial was added and incubated for 5 min according to a previously described technique [36] (Figure 2 and Figure 3) and 50 µL solution calcium chloride (CaCl2) (Galenica Senese, Italy) at a concentration of 5%. The adding of thrombin and/or calcium chloride has been documented to promote the gel formation [37,38]. Glass was used because of its characteristics as a potent platelets activator without the need for anticoagulants, bovine thrombin, or gelling agents. [39].

### 2.2. Mechanical Investigations

**Figure 4:**
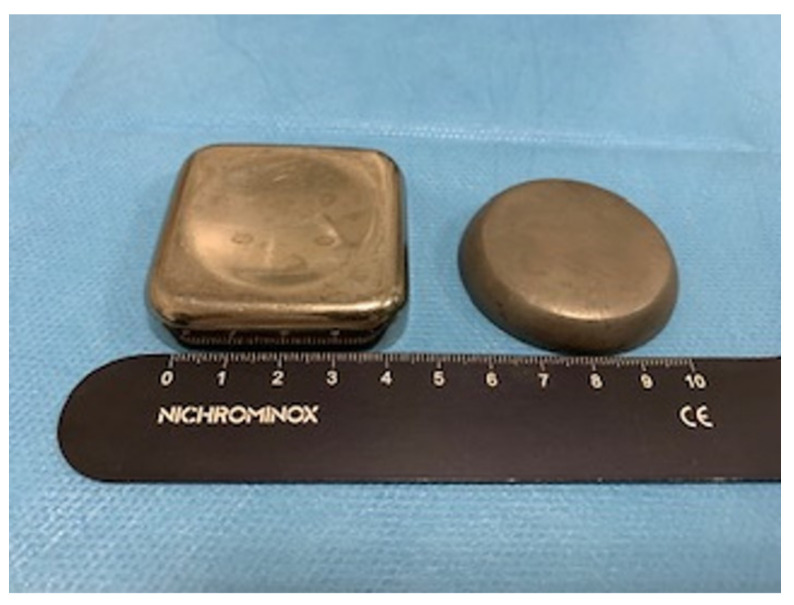
Detail of the mechanical counter-mold used for the APG clots.

A total of twenty APG cylindrical clots, ten APG activated and ten non-activated APG, were obtained. The cylindrical clots were used for the measurement of the mechanical properties and mean value was computed. A dedicated designed mold was acheived in plexiglass to standardize the dimension of fibrin samples according to the same volume, cubic shape, and size. The mold was 2 mm in width, 31 mm in length, and 6 mm at the large ends with a total volume of 104 mm^3^ (Figure 4). 

**Figure 5:**
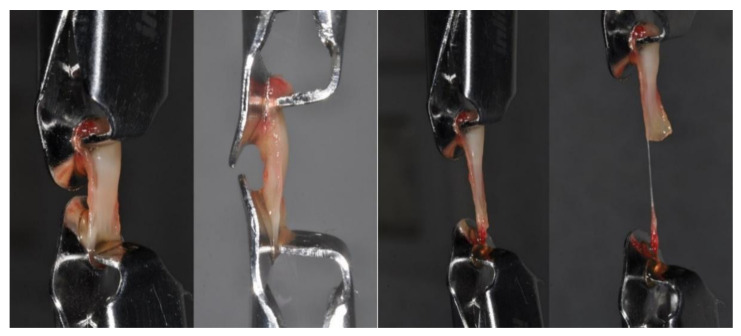
APG during tensile strength under tensile loading.

The tensile strength and elastic modulus tests were applied to determine the behavior of the clots under a tensile loading. Tensile tests were conducted by the traction of the membranes between the device clips with no tension. The specimens were blocked between the clips of the machine without generating any tension. During the investigation, the samples’ traction and mechanical deformation under the applied load were recorded (Figure 5). The tensile strength test was applied to measure the elastic and strength limits. The mechanical characteristics of the study specimens were evaluated by a universal material testing machine (Lloyd 30 K, Lloyd Instruments Ltd., Segensworth, UK) and the study data were obtained by a dedicated software package (Nexigen, Batch Version 4.0 Issue 23, Lloyd Instruments Ltd., Steyning Way, UK). In particular, the tensile test was conducted by a load applied to the samples according to the constant crosshead speed of 1 mm/min on a total of 10 APG clots of activated APG (Group I), and 10 clots of non-activated APG (Group II). The loading was tested longways to the maximum deformation data and the tensile strength data were automatically recorded by the software (Nexigen, Batch Version 4.0, Issue 23, Lloyd Instruments Ltd., Steyning Way, UK). The maximum group strength at sample failure was recorded and tensile strength was calculated by the stress-strain curve recorded by the built-in software. The mechanical test was performed immediately after the molding with no alteration of its composition.

### 2.3. Statistical Evaluation

The statistical analysis was conducted by the statistical software package Graphpad 6 (Prism, San Diego, CA, USA). The data recorded during the experiment were collected by a specially designed worksheet. Given the small sample size, a conservative approach based on nonparametric evaluation by the Wilcoxon signed-rank test was performed to evaluate the mean difference between the two study groups and the level of significance was considered as *p* < 0.05.

The relationships between the tensile strength and Young’s elastic modulus and the and age/gender variables were evaluated by multiple linear regression analysis using the software package Graphpad 6 (Prism, San Diego, CA, USA).

## 3. Results

### Mechanical Characterization

**Figure 6:**
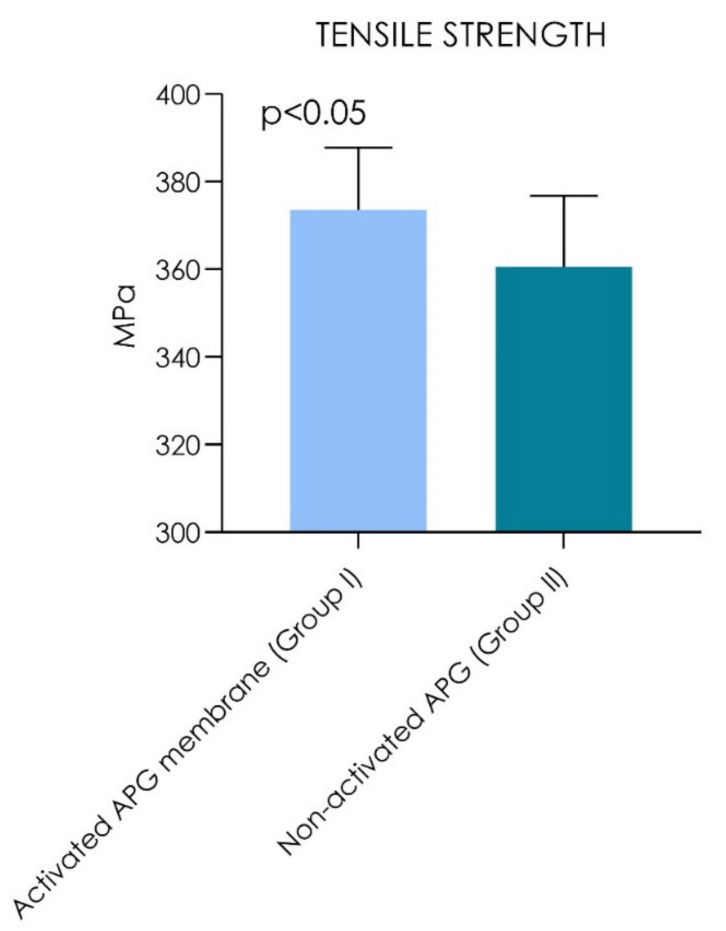
Graph chart of the tensile strength (MPa). A statistically significant difference was detected between the groups during comparison (Wilcoxon signed-rank test, *p* < 0.05).

**Figure 7:**
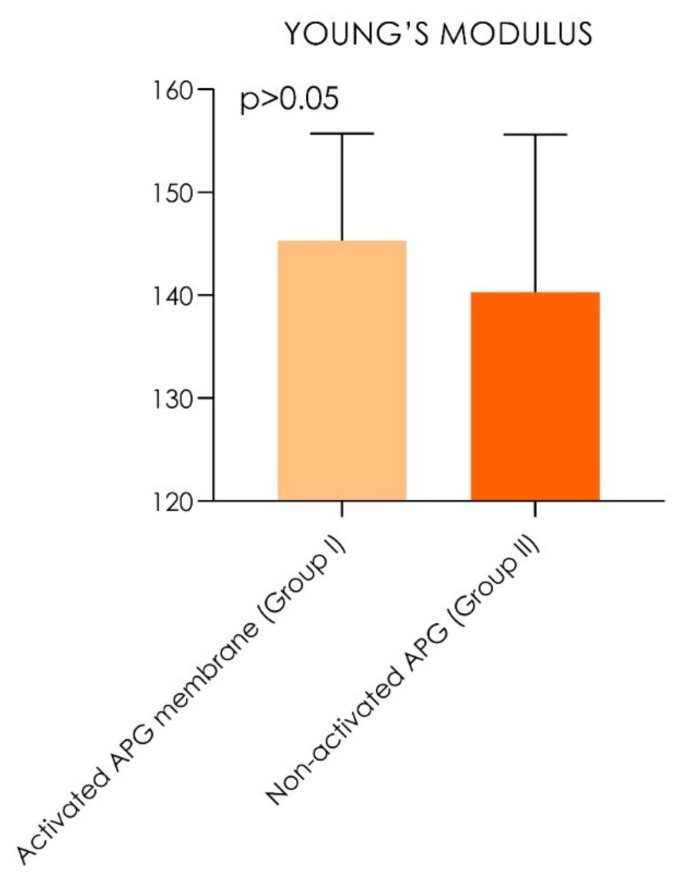
Graph chart of the Young’s modulus. No statistically significant difference was detected between the groups during comparison (Wilcoxon signed-rank test, *p* > 0.05).

The study data were recorded by the machine extensometer by the elastic regime of deformation of the study samples, and the resulting mechanical properties under tensile strength (Table 1) showed that the non-activated APG had a tensile strength of 360.5 ± 16.3 MPa, while APG activated with thrombin and chloride calcium had a tensile strength of 373.5 ± 14.3 MPa (*p* = 0.0020) (Table 1; Figure 6 and Figure 7). 

The samples showed a linear range where the stress was increased proportionally to the mechanical strain. The curve of this region was defined as Young’s modulus, or elastic modulus. The non-activated APG (E = 140.3 ± 15.3 MPa) and activated APG (E = 145.3 ± 10.4 MPa) were similarly elastic under tensile strength. The comparative evaluation of the elastic regime of deformation resistance showed no statistical differences between Group I and Group II (*p* >0.05). Table 2 shows that the tensile strength was not significantly correlated to the age (non-activated APG: *p* = 0.452; activated APG: *p* = 0.443) and gender (non-activated APG: *p* = 0.871; activated APG: *p* = 0.804) when considering both groups’ overall data (*p* < 0.0001). Table 3 reports no significant correlation of elastic modulus with the age (non-activated APG: *p* = 0.401; activated APG: *p* = 0.579) and gender (non-activated APG: *p* = 0.733; activated APG: *p* = 0.874) when considering both groups’ overall data (*p* < 0.0001). The study findings reported that the activated APG group showed a tensile resistance increase of 3.60% and an elastic modulus increase of 3.56% compared with the non-activated APG.

## 4. Discussion

The present investigation highlighted similar mechanical characteristics and strength of non-activated APG and activated APG. To reduce errors, we chose the cylindrical shape to obtain specimens identical in shape, volume, and size. We evaluated the mechanical characteristics of the fibrin clots because they are essential for functions such as stopping bleeding and regenerative procedures. In fact, it is important for these procedures that clots have a material which is highly moldable and mechanically resistant under physiological conditions [35]. In fact, the membranes that are used in bone regeneration procedures should have high mechanical properties to protect and stabilize blood clots and the healing process, especially in the early stages [40]. In literature, the application of an autologous gel with an increased strength consistency is indicated if agglutinated with bone graft, producing increased scaffold stability when positioned into the bone defects [3,7,41]. The combination of calcium chloride and thrombin is able to produce an increase of the mechanical characteristics and a higher new bone formation in vivo [41]. High mechanical resistance of filler or scaffold offer better support against forces from overlying soft tissues and infiltrating cells from the adjacent tissues.

In fact, bone defect healing is sustained by several factors such as osteoconduction, growth factors’ local release, osteoprogenitors’ differentiation and proliferation, and the mechanical stability of the regenerative micro-environment [12,42].

Clinical application of the autologous platelet derivates has been proposed for different regenerative applications due to their biological, colligative, and coagulative properties as membranes, clots, or in liquid form. In literature it was reported that the the platelet growth factor is able to modulate wound healing, stimulating the new angiogenesis and the promotion of cell proliferation and migration [43,44]. Moreover, the platelet growth factor is involved in the sustaining of the soft connective tissues’ renewal, increasing fibroblast activity and collagen synthesis [45,46].

In a previous study, we investigated the mechanical strength of autologus platelet derivates for use in moldable sticky grafts mixed with bone particles [35]. The results showed that, due to the mechanical property of the sticky graft blocks, they can easily be put into bone defects of any shape or size. Thus, the graft for bone regeneration procedures must be of high biocompatibility with host tissue and cells and have strong mechanical properties for maintaining space against the tensile and compressive forces from the overlying tissues. Many studies have investigated the mechanical resistance of fibrin clots, but it is difficult to compare the results because different methodologies were adopted. Alston’s method used a dedicated designed plexiglass to make fibrin specimens with the same volume and size, using a dog bone-shape mold [28]. Adopting a similar protocol, the mold was 2 mm in width, 31 mm in length, and 6 mm at the large ends with a total volume of 104 mm^3^. The authors recorded the tensile strength of leukocyte platelets (L-PRF) (mean value of 0.20 ± 0.06MPa), which was significantly increased compared to the platelet rich growth factor (PRGF) group (value of 0.14 ± 0.07MPa). Ravi and Santhanakrishnan [47] investigated the mechanical properties of three different types of PRF membranes through a PRF box under the compressor and lid for 10 min. The authors recorded the tensile strength of T-PRF (titanium prepared platelet-rich fibrin), obtaining a mean value of 404.61 ± 5.92MPa which was significantly higher than that of A-PRF (362.565 ± 5.15 MPa) and L-PRF, which contained the least tensile strength (290.076 ± 5.68MPa). According to the outcomes of our study, the tensile strength of non-activated APG membranes was similar to that of APG membranes activated with autologous thrombin and calcium chloride. In the present investigation the blood specimens were collected in silica-coated tubes that are known in literature as a potent platelet and clot activator [26,35]. The effectiveness of the present investigation did not highlight an enhancement effect produced by thrombin and calcium chloride addition.

These outcomes could not be compared with other studies because the shape of the specimens was different. Our results may be due to their equal structure, which may be affected by variables such as their differences in polymerization or activation. The methods of polymerization have significant impacts on the mechanical properties of fibrin clots; for example, calcium has an effect on the fibrinogen–fibrin system, which increases the rate of fibrin monomer polymerization [48], generating an immature fibrin matrix and thin fibrils [49].

The activation of the autologous platelet derivates is determined by different events that occur at the moment of preparation, while the platelets’ degranulation is able to produce a release of growth factors from the platelet *α*-granules and the fibrinogen activity is able to produce fibrin matrix organization [44,50]. These events are able to consolidate platelet clot formation and modulate the local release of growth factors [41,51]. The activation process could be accelerated by the adding of thrombin and/or calcium chloride (CaCl_2_); alternatively, spontaneous platelet activation could be produced by local exposure to the native collagenic component naturally present in the connective tissues and in the bone defects [30,39,52]. 

In the present study, we did not observe differences in mechanical resistance when autologous thrombin and calcium chloride was used, but only activated platelets. APG is a totally autologous preparation, and platelet activation and fibrin polymerization are induced by coating the vials in silica. This can be induced either through contact with calcium, present in the collagen of the tissues or present in the biomaterials, or powerful activator platelets released after surgical trauma (ADP, thrombin or collagen). The endogenous activation can stimulate a physiological fibrin polymerization, with a three-dimensional architecture which does not impede chemotaxis and allows a slower and prolonged release of growth factors [33].

The comparison between active and non-activated APG did not reveal statistically significant differences in mechanical resistance. So, despite the similar cellular composition and structure, this suggests that the physicochemical characteristics of the two fibrin clots are similar; the only difference is the presence of activated platelets that should be detected at the ultrastructural level by SEM analysis.

Moreover, the present investigation was limited due to the unidirectionality of the tensile traction applied to the specimens. In fact, clinical presentation is associated also with multidirectional, torsional, and compressive force vectors. In contrast, the present in vitro mechanical simulation took advantage of a highly standardized study model able to provide a repeatable and automated experimental assessment, avoiding any operator biases.

Different studies have reported that the APG clots form a dense fibrin structure, with many ramifications and little spacing observed, especially where platelets are activated [53,54]. A justification of this could be that highly procoagulant cells, for example activated platelets, produce a contractile force among the adjacent filaments, tightening them between the anchoring points of the network [27]. The structural proteins and fibrin network can be scaffolded and can be influenced by the osteoprogenitor cells migrating towards the bone defect. Additionally, fibrin structure, extension, stability, and susceptibility to lysis can influence bone and tissue healing. However, fibrin plays an important role as a scaffold and regulates the release of growth factors. The fibrin clot is a viscoelastic polymer with a highly elastic component for small strains over short times, without an inelastic component [55], while for bigger strains, the stiffness of the fibrin clot increases. This phenomenon, known as strain hardening, is important because it permits fibrin clots to be compliant at normal strain levels and then become stiffer for more deformations and allows clot integrity. Moreover, the clot stiffens and modulates interactions of cells with the fibrin during wound healing. In fact, the cells tend to be different after stress is applied to fibrin by platelet retraction, because the fibrin protofibrils have non-linear elasticity and a linear stretch like other biological polymers [56]. In the present study, we observed thick branching fibers whose elasticity may arise from the bending of the fibers themselves. The fibrin clot is much stiffer for stretching than for flexion and can be reversibly stretched around 4–5 times before rupture. The phenomenon of the fibrin network extending, first aligning then stretching the fibers, determines the larger extensibility. From many results in the literature, it may be concluded that viscoelastic properties are one of the most sensitive measures of the effects of small changes in clotting and structure of activated APG and non-activated APG with thrombin and chloride calcium [57]. In the present study, we used thrombin as an activator because the elastic moduli are greater than for fibrin clots formed with cleavage of only the fibrinopeptides [58]. In fact, Gersh et al. reported not observing the erythrocyte inside of the clot and these data are very important because clot mechanical properties are altered by incorporation of erythrocytes, reducing fibrin ramifications and decreasing fibrin density [59].

## 5. Conclusions

Within the limitations of the present study, the APG membranes showed in vitro useful physical and tensional proprieties, while the activation protocol by autologous thrombin and calcium chloride seems to produce no influence on its mechanical behavior. A larger sample size study and an ultrastructural evaluation of the APG activation is necessary to refine and confirm the effectiveness of the present research.

## Figures and Tables

**Table 1:** Summary of the tensile strength (MPa) and Young’s modulus (Wilcoxon signed-rank test).

	Tensile Strength			Young’s Modulus		
	Mean	SD	95% CI	Mean	SD	95% CI
Activated APG membrane (Group I)	373.5 MPa	(±14.3)	(348.2–372.8)	145.3 MPa	(±10.4)	128.8–151.8
Non-activated APG (Group II)	360.5 MPa	(±16.3)	(362.7–384.3)	140.3 MPa	(±15.3)	132.5–148.1
*p* values	*p* = 0.0020			*p* = 0.40		

**Table 2:** Multiple linear regression analysis for tensile strength.

Multiple Linear Regression for Tensile Strength					
Activated APG membrane (Group I)					
Variable	|t|	*p* value	Estimate	Standard error	95% confidence interval
Intercept	15.56	<0.0001	15.56	22.94	302.7 to 411.1
age	0.95	0.443	0.8926	0.8019	−1.180 to 2.612
Gender (m/f)	0.28	0.804	0.2417	10.64	−27.73 to 22.58
Non-activated APG (Group II)					
Variable	|t|	*p* value	Estimate	Standard error	95% confidence interval
Intercept	13.07	<0.0001	341.6	26.14	279.7 to 403.4
Age	0.8926	0.452	0.8158	0.9140	−1.345 to 2.977
Gender (m/f)	0.2417	0.871	-2.930	12.13	−31.60 to 25.74

**Table 3:** Multiple linear regression analysis for Young’s modulus.

Multiple Linear Regression for Young’s Modulus					
Activated APG membrane (Group I)					
Variable	|t|	*p* value	Estimate	Standard error	95% confidence interval
Intercept	7.687	<0.0001	128.2	16.68	88.78 to 167.7
Age	0.8926	0.579	0.5205	0.5832	−0.8584 to 1.899
Gender (m/f)	0.2417	0.874	−1.870	7.737	−20.16 to 16.42
Non-activated APG (Group II)					
Variable	|t|	*p* value	Estimate	Standard error	95% confidence interval
Intercept	4.993	<0.0001	122.5	24.54	64.50 to 180.6
age	0.8926	0.401	0.7658	0.8579	−1.263 to 2.794
Gender (m/f)	0.2417	0.733	−2.751	11.38	−29.66 to 24.16

## Data Availability

All experimental data to support the findings of this study are available from the corresponding author upon request. The authors have annotated the entire data building process and empirical techniques presented in the paper. The data underlying this article are not freely available by agreement with our partners to protect their confidentiality.
